# DNA vaccination for prostate cancer, from preclinical to clinical trials - where we stand?

**DOI:** 10.1186/1479-0556-10-9

**Published:** 2012-10-09

**Authors:** Sarfraz Ahmad, Paul Sweeney, Gerald C Sullivan, Mark Tangney

**Affiliations:** 1 Cork Cancer Research Centre, BioSciences Institute, University College Cork, Cork, Ireland; 2 Department of Urology, Mercy University Hospital Cork, Cork, Ireland; 3 Department of Urology, Ninewells Hospital Dundee, Dundee, UK

## Abstract

Development of various vaccines for prostate cancer (PCa) is becoming an active research area. PCa vaccines are perceived to have less toxicity compared with the available cytotoxic agents. While various immune-based strategies can elicit anti-tumour responses, DNA vaccines present increased efficacy, inducing both humoural and cellular immunity. This immune activation has been proven effective in animal models and initial clinical trials are encouraging. However, to validate the role of DNA vaccination in currently available PCa management paradigms, strong clinical evidence is still lacking. This article provides an overview of the basic principles of DNA vaccines and aims to provide a summary of preclinical and clinical trials outlining the benefits of this immunotherapy in the management of PCa.

## Introduction

Prostate cancer (PCa) is a major urological problem associated with significant morbidity and mortality [1]. Although the majority of PCa cases are localised to the prostate, nearly one-third of newly diagnosed patients have advanced or metastatic disease [2]. Radical prostatectomy and radical radiotherapy are potentially curative treatment options for localised PCa. For those with locally advanced or metastatic disease, the initial systemic therapy is androgen deprivation therapy. Unfortunately, nearly all patients in this group eventually progress to castration resistant PCa (CRPC). For CRPC, docetaxel based chemotherapy is used as first-line. However, this only provides modest survival benefit (2.4 months) and is associated with significant side effects [3]. Insights into the regulation of immune responses in malignancies have facilitated the emergence of novel immune-based strategies. PCa is an attractive target for vaccination due to its slow growth which can allow sufficient time for immune activation [4]. Additionally, the identification of prostate tumour associated antigens (that are recognised by T cells) has created the opportunity to develop novel immune based therapeutic approaches. Several tumour associated antigens have been identified, including prostate specific antigen (PSA), prostatic acid phosphatase (PAP), prostate stem cell antigen (PSCA) and prostate specific membrane antigen (PSMA) [5]. These antigens are largely prostate specific and their expression is strongly upregulated in PCa, both locally and at metastatic sites [6,7]. This makes these antigens a viable target of active immunotherapy and can be used as DNA vaccines. Furthermore, PSA-specific cellular immune responses have been detected in some PCa patients and in normal individuals, suggesting that tolerance towards this antigen may be broken [8]. These observations led to development of a variety of vaccines for PCa in pre-clinical and clinical trials. These vaccines can be broadly classified as whole cell vaccines [9], protein/peptide based vaccines [10,11], viral vector based vaccines [12], dendritic cell vaccines [13] and naked DNA vaccines [14,15].

Like other forms of targeted therapy, cancer vaccines hold the promise of achieving cancer control without inducing overt toxicity. The focus of extensive research for PCa vaccine has led to the approval of the first therapeutic vaccine (sipuleucel-T) by the FDA [16]. The sipuleucel-T is an autologous antigen presenting cell (APC) based and antigen-targeted immunotherapeutic innovation for men with CRPC [16]. The success story of the sipuleucel-T encouraged researchers to explore other agents/strategies to activate immune system against PCa. Gene therapy (including DNA vaccines) is a realistic prospect for the treatment of prostate and other cancers, and involves the delivery of genetic information to the patient to facilitate the production of therapeutic proteins. A DNA vaccine consists of tumour specific/associated antigen and additional immune-stimulatory factors cloned into a bacterial plasmid downstream of an appropriate eukaryotic promoter for strong and stable expression. DNA vaccination can induce effective anti-tumour responses against various malignant cells and provides an attractive strategy for the management of PCa [17-19].

## Why DNA vaccines?

DNA immunisation can efficiently stimulate humoural and cellular immune responses to protein antigens. This strategy has been used successfully for infectious diseases [20] and potentially can be applied for malignant conditions. There are several advantages associated with DNA vaccines:

Various groups have demonstrated tumour protection using DNA immunisations in several pre-clinical cancer models [21-26].

Gene sequences can be manipulated easily to provide multiple potential epitopes that stimulate both humoural and cellular immunity [22,27-29].

DNA vaccines generally skew the immune system toward the desired T-helper1 (Th1) response. This is most likely because plasmid DNA contains unmethylated CpG motifs, which have been shown to be a very potent immunological adjuvant [30,31].

DNA immunisation is safe in humans [14,32] and can induce antigen-specific immune responses [30,33].

DNA vaccines can be produced readily at a large scale [34].

## Mechanisms of action

### Anti-tumour immune responses

For malignant diseases, immunological therapies fall into two general categories; active and passive immunotherapy. Active immunotherapy attempts to directly elicit tumour-specific host immune responses that control or eradicate tumours. In contrast, passive immunotherapy involves the direct administration of effector molecules, such as cytokines or monoclonal antibodies. These molecules promote the development of anti-tumour responses, directly killing tumour cells, or inhibit cell invasion and angiogenesis [35]. The nature of the host immune response that can control tumour growth has been the focus of many studies. With few exceptions, the most effective anti-tumour immune responses in animal models have depended on the efficient generation of Th1 cell immunity, characterised by strong cytotoxic T lymphocyte (CTL) responses [27,28,36]. This is supported by observations in humans that progressive disease is characterised by an anti-tumour T-cell response [37] as well as by clinical evidence that Th1 T cells can control and eliminate metastatic disease [28,38]. B cell effector functions are another important component of this anti-tumour immune response (see below).

### Process of DNA vaccination

A typical DNA vaccine consists of a transgene that encodes the sequence of a target protein (e.g. PSA, PSMA etc.) under the control of a eukaryotic promoter. Various modalities exist for delivering such DNA to appropriate cells (see below). After uptake of the plasmid, the target protein is produced within the cell and processed into small antigenic peptides by host proteases. The peptides then enter the lumen of the endoplasmic reticulum (E.R.) by membrane-associated transporters. In the E.R., the peptides bind to Major Histocompatability Complex I (MHC I) molecules. These peptides are presented on the cell surface in the context of MHC I. Subsequently CD8^+^ CTL are stimulated resulting in cell-mediated immunity. CTLs cause tumour destruction through both cytolysis of malignant cells and non-cytolysis mechanisms such as cytokines production.

**Figure 1:**
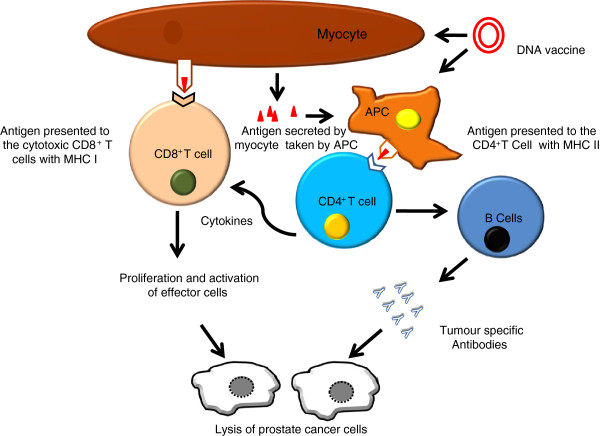
**Mechanism of action of DNA vaccine after intra muscular (i.m.) plasmid delivery.** Transfected muscle cells produce the antigen expressed on the plasmid. This antigen is expressed to cell surface with MHC I and presented to cytotoxic CD8^+^ T cells (Cell mediated Immunity). Antigen is also excreted by the muscle, which is phagocytosed by the professional Antigen Presenting Cells (APC), usually Dendritic cells. A small proportion of DNA vaccine is also taken up directly by APC and the encoded antigen can then be processed and presented endogenously (Humoural Immunity).

The foreign protein on the plasmid can also be presented by MHC II pathway by APC which elicit CD4^+^ helper T cells responses. These CD4^+^ T cells can recognise the peptides generated from the exogenous proteins that were endocytosed or phagocytosed by APC, then degraded to peptide fragments and loaded onto MHC II molecules. Depending on the type of CD4^+^ T cell that binds to the complex, B cells are activated and antibody production is stimulated. This is the same manner in which traditional vaccines work [39]. Vaccine-elicited antibodies can mediate direct effects against tumour cells by fixing complement or facilitating antibody-dependent cellular cytotoxicity. Overall, stimulation of both the T and B cell arms of the immune system mediates synergies and creates a large pool of effectors cells to control tumour growth and induce generation of memory cells (Figure 1).

## Methods of DNA delivery

**Figure 2:**
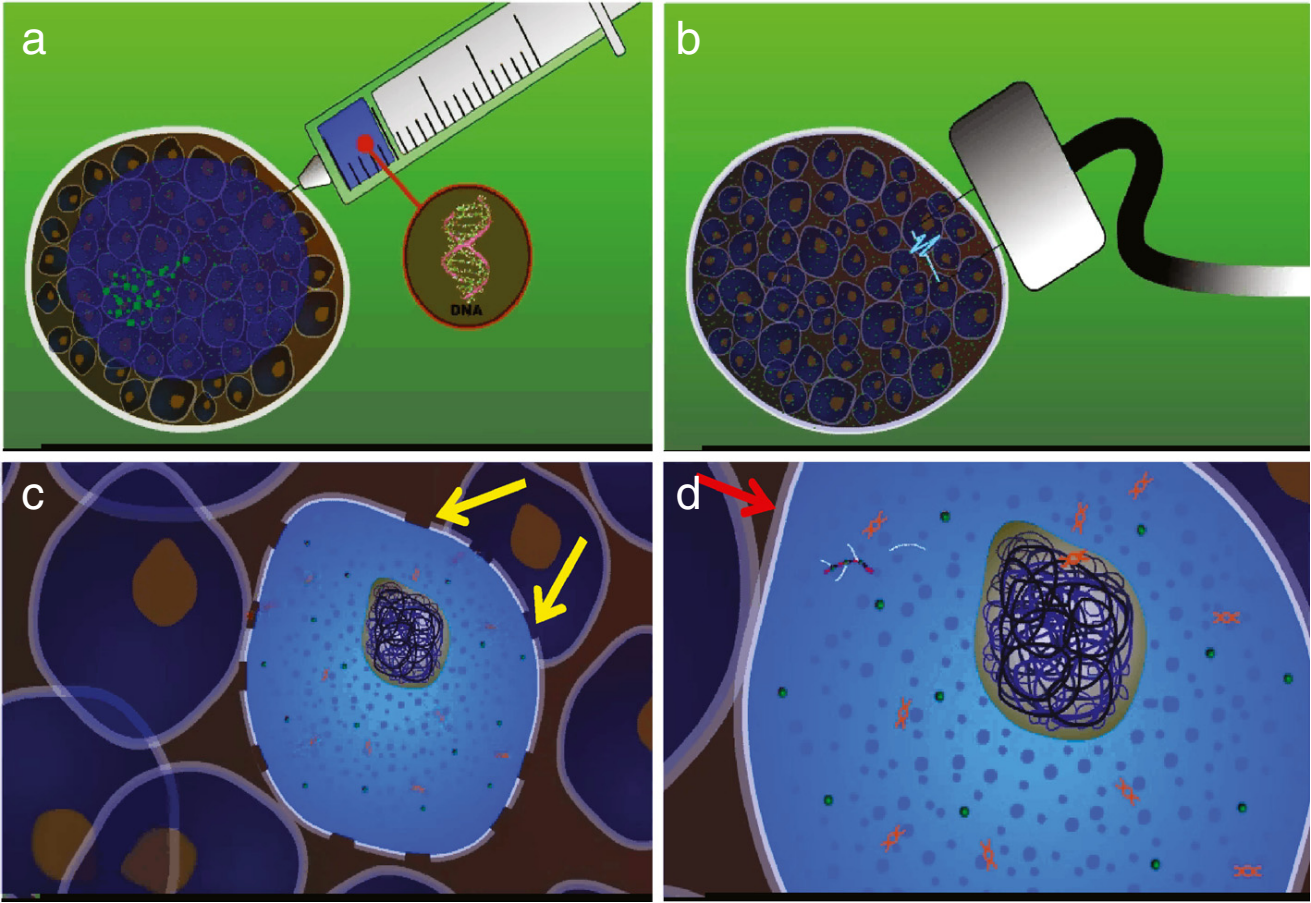
**Schematic representation of Electroporation mediated transfection. a**) Intra-muscular plasmid injection. **b**) Electroporation. **c**) Transient increased permeability of cell membrane (yellow arrows) results in transfer of the plasmid into the cell. **d**) Cell membrane return to resting membrane (red arrow) and gene transfection results in production of mRNA and hence specific protein.

Transfection of the host cell with the plasmid is a limiting step for a successful DNA vaccination. Most commonly, the DNA vaccines are given by intramuscular (i.m.) or intradermal (i.d) injections. To facilitate the gene delivery, various methods have been reported including viral, liposomal, bacterial, ultrasound and electroporation (EP), as well as approaches involving *ex vivo* transfection/transduction of cells (e.g. APC) [40]. To avoid safety issues such as immune response and cytotoxicity associated with viruses and liposomal transfection, physical methods (ultrasound, EP etc.) have been widely used for either *in vivo* or *ex vivo* gene delivery. *In vivo* EP has emerged as a potent method for DNA vaccine delivery and significantly improves transfection efficiency of naked plasmid DNA [41]. EP driven DNA vaccination increases antigen expression by increasing transfection efficiency (Figure 2) and is accompanied by local tissue injury and inflammation [42]. Hence, the outcomes of EP mediated vaccination are dramatic enhancement of humoural and cellular immunity [43,44].

## Preclinical studies

Numerous preclinical studies have been carried out to demonstrate the efficacy of DNA vaccines in animal models of PCa. The following vital issues regarding successful vaccines were addressed in these preclinical trials;

Induction of immune response/type of immune activation

Optimisation of vaccine dose/schedule

Mode of delivery

Breaking tolerance to self-antigen

Systemic responses /control of metastatic disease

Need for adjuvant to vaccines

Transferable immunity

### Prostate-specific antigens examined

Roos *et al.*[45] showed for the first time that a PSA DNA vaccine can induce anti-tumour immunity *in vivo*. They investigated a DNA vaccination strategy to immunise mice by inducing PSA-specific cellular responses. A plasmid expressing PSA, alone or in combination with plasmids coding for granulocyte-macrophage colony-stimulating factor (GM-CSF) and/or IL-2, were used. It was found that the DNA vaccine induced PSA-specific cytotoxic T lymphocytes and when co-injected with GM-CSF and IL-2 it can protect mice against a PSA-expressing tumour challenge. This demonstrated that immunisation with a PSA DNA vaccine can evoke PSA-specific cellular immunity. The ability of the DNA vaccine to stimulate both humoural and cellular immune responses was demonstrated by Kim *et al.*[24]. They observed a strong and persistent antibody response against PSA for at least 180 days following immunisation. Additionally, significant T helper cell proliferation was also detected against PSA. Furthermore, immunisation with PSA plasmid induced CD8^+^ T cell-restricted cytotoxic T cell response against tumour cell expressing PSA.

PSMA is a 750 amino acid surface protein expressed primarily in prostate epithelium, Most reported antibodies to PSMA apparently recognise epitopes in the residue 43–570 region of the extra cellular domain, and upon binding are rapidly removed from the cell surface by internalisation. This would potentially limit their ability to mediate Fc-dependent cytotoxicity. Kuratsukuri *et al.*[46,47] used a model system to target a defined region of the extra cellular domain of PSMA. Their results suggested that vaccination with plasmid expressing extra cellular domain of PSMA induced adaptive humoural activity, which was directed against the extracellular region of human PSMA and can significantly inhibit human PCa growth in athymic mice.

Viral delivery of vaccine antigens is an active research area. However, a potential difficulty with viral-based immunisations is that immune responses elicited to the viral vector might limit the possibility of multiple immunisations. Johnson *et al.*[48] investigated a DNA vaccine encoding PAP, to elicit antigen-specific CD8^+^ T cell immune responses. In their study, Lewis rats were immunised with either a plasmid DNA-based (pTVG-HP) or Vaccinia-based (VV-HP) vaccine each encoding human PAP (hPAP). They observed Th1-biased immune response (as indicated by proliferating PAP-specific CD4^+^/CD8^+^ cells and IFN- *γ* production) in rats immunised with a DNA vaccine encoding hPAP. Immunisation with Vaccinia virus (encoding hPAP) could not induce PAP-specific response, unless boosted with a heterologous vaccination scheme. Furthermore, they also established that multiple immunisations with a DNA vaccine encoding the rat PAP homologue (pTVG-RP) could overcome peripheral self-tolerance against rat PAP (rPAP) and generate a Th1-biased antigen-specific CD4^+^ and CD8^+^ T cell response. In separate experiments, this DNA vaccine has not shown significant toxicities in terms of animal weights, histopathology, haematological changes, or changes in serum chemistries [49]. The vaccine was found to be effective in eliciting PAP-specific CD4^+^ and CD8^+^ T cells, predominantly Th1 in type, in all immunised animals at all doses and numbers of immunisations. PAP-specific IgG were detected in a dose-dependent fashion, with titres increasing after multiple immunisations.

### Optimisation of DNA vaccination

In preclinical trials different doses and vaccination schedules are investigated with variable results however, there is lack of consensus on these issues. Ahmad *et al.* have shown that the a DNA vaccine encoding human PSA significantly delayed the appearance of tumours and resulted in prolonged survival of the animals [50]. Additionally, a four-dose vaccination regimen provided optimal immunological effects and co-administration of synthetic CpG enhanced the tumour protective responses [50]. Furthermore, these immune responses were tumour specific and were transferable in adoptive T cell transfer experiments [50]. A DNA vaccination has the potential to break tolerance to self antigen [34,48,51]. A PSCA DNA vaccine when delivered by i.m. EP [51] or orally administered bacteria, [52] resulted in induction of anti-tumour immune responses against PSCA expressing subcutaneous tumours and metastatic deposits. There was activation of Th-1 type immunity against PSCA, indicating the breaking of tolerance to a self-antigen [51]. This immunity was tumour specific and was transferable by adoptive transfer of splenocytes [51].

PSMA is present in both secretary form and in prostate cells. Mincheff *et al.*[53] evaluated two plasmid DNA vaccines, encoding either PSMA products that are retained in the cytosol and degraded in the proteasome (tVacs; hPSMAt), or secreted proteins (sVacs; hPSMAs) for stimulation of cytotoxic cell or antibody responses. They observed that immunisation with both vectors led to generation of cell cytotoxicity, provided GM-CSF was administered with the vaccine. Spleen cells from animals immunised with hPSMAt demonstrated stronger cytotoxicity to the target cells. Interestingly, priming with a vector that encoded a xenogeneic protein (hPSMAt; ‘xenogeneic’ construct) and boosting with a vector that encoded an autologous protein (rPSMAt; ‘autologous’ construct) gave the best protection against tumour challenge. Immunisation with tVacs did not lead to formation of antibodies to the target protein, while immunisation with sVacs or with the protein did (mixed Th1-Th2 isotype). But, priming with tVacs and boosting with protein also resulted in the antibodies from the cytotoxic Th1 isotype. Hence, the best strategy to obtain a strong cellular cytotoxic response seems to be gene-based vaccinations with tVacs, priming with the ‘xenogeneic’ and boosting with the ‘autologous’ constructs.

While DNA vaccine injections, either i.m. or i.d., have been used successfully in many trials, it is still to establish which route is better. To enhance the efficacy of DNA vaccine against PCa, Roos *et al.*[54] have demonstrated that i.d. DNA vaccination, followed by two sets of electrical pulses of different length and voltage, can effectively induce PSA-specific T cells response. Ahmad *et al.* have reported successful DNA vaccination following i.m. injection coupled with EP. [50,51] These studies indicate that EP significantly enhances transfection and hence immune activation.

## Clinical trials

Preclinical studies provided encouraging evidence of enhanced immune responses and tumour protection by DNA vaccine in animal models of PCa. The success achieved in these studies resulted in exploration of application of DNA vaccine in PCa patients. To our knowledge, no naked DNA vaccine has been used in a randomised clinical trial to date [55]. However, DNA vaccines have been used in phase I/II clinical trials including patients with PCa (Table 1). Some of these clinical trials are discussed below.

**Table 1:** Summary of prostate cancer DNA vaccination clinical trials

**References**	**Antigen /+− co stimulatory molecules**	**No. of patients/ patient’s characteristics**	**Type of study**	**Route of vaccination**	**Immunological responses**	**Adverse effects**	**PSA response**
[14]	Extracellular human PSMA & CD_86_ into separate expression vectors (PSMA & CD_86_ ), and into a combined plasmid (PSMA/CD_86_)	26	Phase I/II	i.d.	- All patients who received initial inoculation with viral vector followed by PSMA plasmid boosts showed immunisation. In contrast, with PSMA and CD_86_ plasmids, only 50% were immunised.	-	-
	+ Expression cassette from PSMA plasmid into a replication deficient adenoviral expression vector				- Of the patients who received PSMA & GM-CSF, 67% were immunised. However, PSMA/CD_86_ & GM-CSF vaccination immunised all recipients.		
[15]	Plasmid vector expressing PSA & GM-CSF/IL-2	9 CRPC	Phase I	i.m, i.d.	PSA-specific cellular immune response (measured by IFN- *γ* & anti-PSA IgG levels) were detected in highest dose cohort of patients.	- Systemic effects; running nose, fatigue, myalgia, chills and fever (*n = 6*).	- Drop in PSA (*n=3*).
						- At the injection site; erythema, swelling, induration, itching, pain, urticaria (*n = 7*).	- Increase in PSA (*n= 5*).
[56]	Vaccine encoding a domain of fragment C of tetanus toxin fused to a tumour-derived epitope from PSMA	5 patients / dose level	Phase I/II,	i.m. or i.m. + EP	Delivery of DNA+EP at all five vaccinations resulted in activation of humoral immunity.	- Mild pain at injection site.	-
		Recurrent PCa				- EP did not add toxicity.	
[57]	Vaccine encoding PAP co-administered with GM-CSF	22 Stage D_0_ PCa	Phase I/IIa	i.d.	- Three of 22 patients developed PAP-specific IFN-*γ* secreting CD8^+^ T-cells. While 9 (41%) patients developed PAP-specific CD4^+^ and/or CD8^+^ T-cell proliferation.	No significant adverse events	PSA doubling time increased from a median 6.5 months per treatment to 8.5 months on-treatment & 9.3 months in one year post treatment.
					- Antibody responses to PAP were not detected.		

*PSMA* Prostate Specific Membrane Antigen, *CD*_*86*_ Cluster of Differentiation 86, *i.d* Intradermal, *i.m* Intramuscular, *PSA* Prostate Specific Antigen, *GM-CSF* Granulocyte-macrophage colony-stimulating factor, *IL2* Interleukin 2, *CRPC* Castrate Resistant Prostate Cancer, *IFN-γ* Interferon Gamma, *PCa* Prostate Cancer, *PAP* Prostate Acid Phosphatase.

In a phase I trial, Pavlenko *et al.*[15] investigated the feasibility, safety and immunogenicity of a DNA vaccine (pVAX/PSA) in patients with CRPC. Cytokines, GM-CSF and IL-2, were also used as vaccine adjuvants. The results of this trial demonstrated that DNA vaccination with a PSA-coding plasmid vector, given with GM-CSF and IL-2, is safe and can induce cellular and humoural immune responses against PSA. However, a dose–response was observed with regard to induction of a PSA-specific immune response. Interestingly, in this trial, two patients that developed cellular immune response to PSA exhibited stabilisation of disease. While only one of six that did not develop PSA-reactivity showed clinical stabilisation.

Mincheff *et al.*[14] performed phase I/II trials to determine the safety of the PSMA vaccine after repeated i.d. injections. Twenty-six patients with PCa were entered into this toxicity-dose escalation study. Immunisations were performed i.d. at weekly intervals. Doses of DNA between 100 and 800 μg and of recombinant virus at 5×10^8^ PFUs per application were used. They observed no immediate or long-term side effects following immunisations. All patients who received initial inoculation with the viral vector followed by PSMA plasmid boosts showed the development of a delayed-type hypersensitivity reaction after the PSMA plasmid injection. In contrast, to the patients who received a PSMA plasmid and CD86 plasmid, only 50% showed signs of successful immunisation. Of the patients who received PSMA plasmid and soluble GM-CSF, 67% were immunised. However, all patients who received the PSMA/CD86 plasmid and sGM-CSF became immunised. The patients who did not immunise during the first round were later successfully immunised after a boost with the viral vector. Further to this study, Todorova *et al.*[58] characterised the humoural immune response against PSMA in PCa patients. They demonstrated that PSMA is a target for humoural immune response induced by gene-based PSMA vaccination. It is also proposed that detection of anti-PSMA antibodies by immunoblot analysis and by indirect immunofluorescence could be used to monitor the vaccination effects. These results were quite encouraging, proving evidence of immune activation with different vaccination regimens. However, the heterogeneity of the medical status and the presence of concomitant hormone therapy do not permit unequivocal interpretation of the data with respect to the effectiveness of the therapy. However, several responders, as evidenced by a change in the local disease, distant metastases, and PSA levels, were identified in this cohort.

Low *et al.*[56] evaluated the use of EP to deliver a novel DNA vaccine (p.DOM-PSMA_(27)_). This vaccine encodes a domain of fragment C of tetanus toxin to induce CD4^+^ T cell help, fused to a tumour-derived epitope from PSMA for use in HLA-A2^+^ patients with recurrent PCa. In this open label phase I/II, two-arm, dose escalation trial, the DNA vaccine was delivered either by i.m injection or by i.m. injection followed by EP. Three vaccinations were given at 0, 4, and 8 weeks, with booster doses at 24 and 48 weeks. In 20 patients with first two dose cohorts, EP did not appear to add toxicity to the vaccination apart from brief and acceptable pain at injection site. They also observed highest level of humoural responses with DNA+EP strategy and these responses persisted to 18 months of follow-up. These data favour EP as a potent method for stimulating humoural responses induced by DNA vaccination in humans.

McNeel *et al.*[57] conducted a phase I/IIa trial with a DNA vaccine encoding human PAP in patients with stage D_0_ PCa. Twenty-two patients were treated in a dose-escalation trial with 100 μg, 500 μg, or 1,500 μg plasmid DNA, co-administered i.d. with 200 μg GMC-SF as a vaccine adjuvant, six times at 14-day intervals. All of these patients were observed for one year after the treatment. They did not observe any significant adverse events. Three of 22 patients developed PAP-specific IFN-*γ* secreting CD8+ T-cells immediately after the treatment course. While nine (41%) out of 22 patients, developed PAP-specific CD4+ and/or CD8+ T-cell proliferation. However, no humoural response (antibodies against PAP) was detected. Overall, the PSA doubling time was observed to increase from a median 6.5 months pre-treatment to 8.5 months on-treatment (*P* = 0.033), and 9.3 months in the 1-year post-treatment period (*P* = 0.054). This study provided 12 months follow up data and demonstrated that the DNA vaccine is safe, elicits an antigen-specific T-cell response, and may be associated with an increased PSA doubling time.

## Conclusions and future directions

DNA vaccination for PCa is at a crucial developmental stage. The ultimate goal of any given immunotherapy including DNA vaccination is eradication of each and every cancer cell from the patient. However, this goal may be hard to achieve. DNA vaccination is a step forward in achieving immune eradication of PCa. Induction of tumour-specific T cell activation has been demonstrated with PCa DNA vaccination in both preclinical and clinical trials. However, in clinical settings, limited success has been seen in terms of tumour regression and survival. This vaccine failure may be attributed to several potential tumour escape mechanisms such as defects in antigen presentation, production of immunosuppressive substances, T cell dysfunction, and the presence of regulatory T cells [59-61]. Additionally, there is paucity in the literature of advanced clinical trials to evaluate the role of DNA vaccination in PCa. Furthermore, in the available trials there is currently a lack of long-term follow up. Ideally, the availability of data from randomised clinical trials featuring robust end points such as biochemical response, progression free and overall survival will provide categorical evidence for DNA vaccination’s potential.

In order to provoke an immune response, a tumour vaccine should not only maximise antigen-specific signals, but should also provide the necessary “co-stimulatory” environment. One approach is to include lymphokines (GM-CSF, IL-12, IL-15) or include tumour cell expression of membrane bound molecules (CD80, CD86) [62]. Furthermore, doses and schedules need optimisation; however it is clear that the immune responses depend on a primary vaccination followed by booster vaccination(s). Some studies have suggested that the best strategy for achieving an intense immune response can be priming with naked DNA followed by boosting with a viral vector [14,63]. The use of viral vectors can enhance the immunogenicity of the vaccine due to the adjuvant properties of some of the viral products. However, naked DNA immunisation offers several potential advantages over viral mediated transduction. Among these are the inexpensive production and the inherent safety of plasmid vectors, as well as the lack of immune responses against the carrier [63].

Although the current evidence suggest that the DNA vaccines can induce immune activation resulting in PCa control, certain areas still need to be explored such as selecting the ideal antigen, identifying suitable stages of PCa for vaccine therapy, optimum dosage/schedule and adjuvant agents. Evidence suggests that it is unlikely for a single therapy to achieve the goal of curing PCa especially patients with CRPC. However, better understanding of immunobiology of PCa will lead to pathways for development of novel therapies. The perceived difficulty in translating the effectiveness of DNA vaccination from small animals to human subjects appears to have been overcome by use of EP [42]. Current data are from early stage studies and further research in this field is essential to determine the place of DNA vaccination as an adjuvant to surgery, radiotherapy and chemotherapy.

## Competing interests

The authors declare that they have no competing interests.

## Authors’ contributions

SA carried out literature search, interpreted the data and drafted the manuscript. MT, PS and GCOS helped with data interpretation and drafting the manuscript. All authors read and approved the final manuscript.
